# Ovine multiparity is associated with diminished vaginal muscularis, increased elastic fibres and vaginal wall weakness: implication for pelvic organ prolapse

**DOI:** 10.1038/srep45709

**Published:** 2017-04-04

**Authors:** Stuart Emmerson, Natharnia Young, Anna Rosamilia, Luke Parkinson, Sharon L. Edwards, Aditya V. Vashi, Miranda Davies-Tuck, Jacinta White, Kirstin Elgass, Camden Lo, John Arkwright, Jerome A. Werkmeister, Caroline E. Gargett

**Affiliations:** 1 The Ritchie Centre, Hudson Institute of Medical Research, Clayton, Victoria, 3168, Australia; 2 Monash University, Department of Obstetrics and Gynaecology, Clayton, Victoria, 3168, Australia; 3 Monash Health, Clayton, Victoria, 3168, Australia; 4 School of Computer Science, Engineering and Mathematics, Flinders University, Bedford Park, South Australia 5042, Australia; 5 CSIRO Manufacturing, Clayton, Victoria, 3168, Australia; 6 MicroImaging, Hudson Institute of Medical Research, Clayton, Victoria, 3168, Australia

## Abstract

Pelvic Organ Prolapse (POP) is a major clinical burden affecting 25% of women, with vaginal delivery a major contributing factor. We hypothesised that increasing parity weakens the vagina by altering the extracellular matrix proteins and smooth muscle thereby leading to POP vulnerability. We used a modified POP-quantification (POP-Q) system and a novel pressure sensor to measure vaginal wall weakness in nulliparous, primiparous and multiparous ewes. These measurements were correlated with histological, biochemical and biomechanical properties of the ovine vagina. Primiparous and multiparous ewes had greater displacement of vaginal tissue compared to nulliparous at points Aa, Ap and Ba and lower pressure sensor measurements at points equivalent to Ap and Ba. Vaginal wall muscularis of multiparous ewes was thinner than nulliparous and had greater elastic fibre content. Collagen content was lower in primiparous than nulliparous ewes, but collagen organisation did not differ. Biomechanically, multiparous vaginal tissue was weaker and less stiff than nulliparous. Parity had a significant impact on the structure and function of the ovine vaginal wall, as the multiparous vaginal wall was weaker and had a thinner muscularis than nulliparous ewes. This correlated with “POP-Q” and pressure sensor measurements showing greater tissue laxity in multiparous compared to nulliparous ewes.

POP is the herniation of pelvic organs into the vaginal cavity. Symptoms are bladder, bowel and sexual dysfunction, including incontinence which severely affects the quality of life of affected women^1^,^2^,^3^. POP affects 25% of all women in the USA and Western countries, and is particularly prevalent in post-menopausal women^4^,^5^. The main risk factors are vaginal delivery and ageing, in addition to obesity contributing to POP recurrence^6^,^7^. A genetic predisposition for developing POP appears to involve genes regulating collagen and elastic fibre synthesis^8^,^9^,^10^. Despite the prevalence of POP, little is understood about the underlying mechanisms affecting the integrity of the vaginal wall that leads to POP. For example, little is known about the effect of parity on vaginal wall structure and function and how these change in women who develop POP. Animal models reflecting human POP are important in developing new treatments for POP.

We are developing a tissue engineering approach for treating POP with the goal of using an ovine preclinical vaginal surgery model to evaluate a new polyamide/gelatin composite scaffold seeded with endometrial mesenchymal stem cells^11^,^12^. Similar to women, ewes develop spontaneous POP^13^,^14^, but the pathophysiological mechanisms that underpin POP are not well understood. We have therefore developed a novel pressure sensor device to detect changes in vaginal wall pressures along the length of the anterior and posterior walls to better diagnose weakened regions that might be associated with POP^15^. In addition, we have adapted the pelvic organ prolapse quantification (POP-Q) system, a clinical score of POP for assessing the ewe vagina^16^. The POP-Q measures vaginal tissue displacement at four points within the vaginal wall which are associated with POP; points A and B on the anterior wall (Aa, Ba), which are associated with anterior vaginal wall POP (cystocele), and points A and B posterior (Ap, Bp)^17^ which is the region vulnerable to posterior vaginal wall POP (rectocele and enterocele, respectively). In ewes, this system measures how far the tissue can be extended from its resting position for 3 of the POP-Q points using traction rather than during Valsalva manoeuver as is performed in women. Higher measurements indicate greater vaginal weakness and greater potential for developing POP.

The histological structure of human and ovine vaginal tissue is similar, comprising an epithelium, collagen-rich lamina propria and a muscularis containing smooth muscle^18^. Changes to the composition of these layers and their relative contribution to POP susceptibility is not well understood^19^. Neither is it known how such changes are reflected in POP-Q measurements or vaginal wall weakness. A relationship between extracellular matrix proteins (ECM) and POP has been identified, with a high content of immature collagen III associated with POP^20^. While collagen type I and III have been shown to increase with prolapse, others have suggested that the ratio of collagen type III to type I decreases^21^. Irrespective, it appears that the collagen remodels with the onset of prolapse, due to increased expression of matrix metalloproteinases (MMP)-2 and -12^22^. This is further exacerbated during the progression of prolapse by an increase in other matrix metalloproteinases, active MMP-9 and a decrease in fibulin 5, essential for elastogenesis^23^. There is also a decrease in the transcriptional regulator, Homeobox protein Hox-A13 (*HOXA13*), which controls the expression of ECM associated genes involved in elastic fibre homeostasis^24^ in vaginal tissue^22^. Elastic fibres play an important role in the mechanical properties of the vaginal wall, allowing the tissue to extend and return to its original shape without damage^25^ particularly during pregnancy^26^. Disruption of elastic fibre synthesis by the absence of lysyl oxidase like 1 (LOXL1) or knock out of Fibulin 5^23^, an essential elastic fibre binding protein required for synthesis, contributes to spontaneous POP in mice^23^,^27^.

Smooth muscle provides mechanical strength to tissue and viscoelasticity to the vagina, allowing for expansion during intercourse and vaginal delivery. There is a reduction in cross-sectional area of nonvascular vaginal smooth muscle in both the posterior and anterior wall in women with POP^28^,^29^,^30^. However the mechanism involved is not well understood^31^. A reduction in smooth muscle content of the vaginal muscularis may impact its biomechanical properties.

The purpose of this study was (1) to analyse “POP-Q” and pressure sensor measurement differences in ovine vagina and (2) to correlate these data with the collagen, elastic fibre and smooth muscle content and biomechanical properties of ovine vaginal tissue to determine if a relationship exists between ECM and smooth muscle content of the vagina, its biomechanical properties and susceptibility to POP by parity. We hypothesised that with increasing parity, ECM proteins and smooth muscle content decreases and is associated with altered biomechanical properties of ovine vaginal tissue. These altered properties will relate to vaginal tissue weakness, reflected in abnormal “POP-Q” measurements and changes in pressure distribution along the ovine vaginal wall.

## Results

### Vaginal weakness identified in multiparous ewes by “POP-Q” and pressure sensor measurements

**Figure 1:**
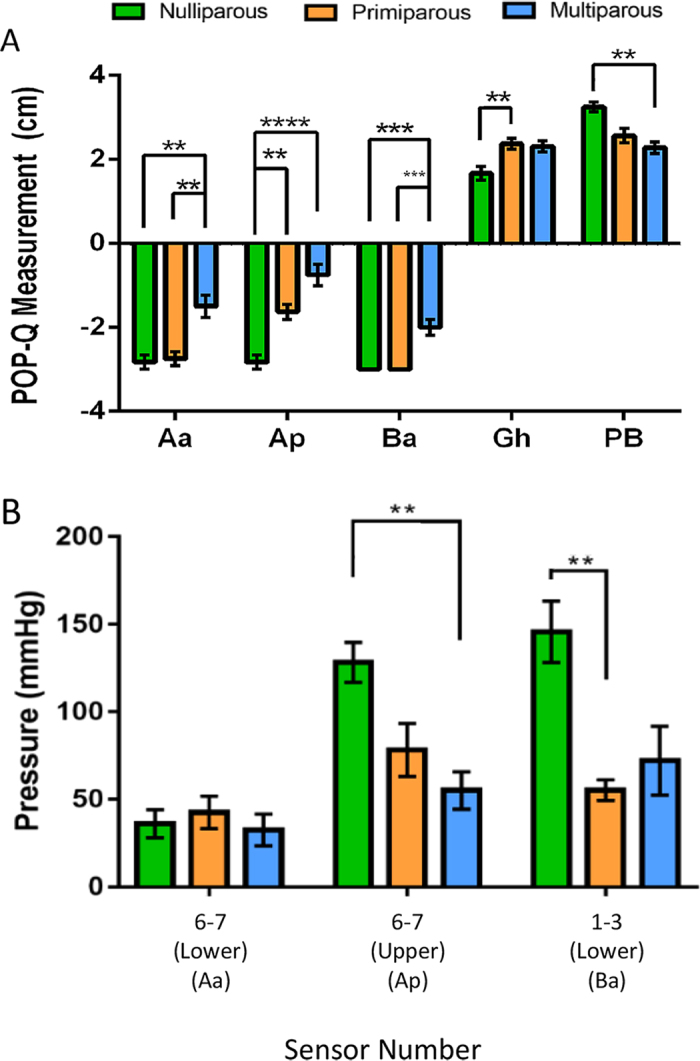
Modified POP-Q and pressure sensor measurements identify vaginal weakness in multiparous ewes. (**A**) “POP-Q” measurements in ovine nulliparous (green bars, n = 6), primiparous (orange bars, n = 8) and multiparous vaginal tissue (blue bars, n = 8). POP-Q points Aa and Ba are taken on the anterior wall 3 cm from the muco-cutaneous junction and 3 cm above the urethra (located in the vaginal wall), Ap 3 cm from the posterior muco-cutaneous junction. Gh; Genital hiatus; Pb, perineal body. (**B**) Pressure sensor measurements were taken from sensors detecting pressure in regions equivalent to Aa (anterior, distal sensors 6–7 on the lower blade), Ap (posterior, distal sensors 6–7 on the upper blade) and Ba (anterior, proximal sensors 1–3 on the lower blade). Data are mean +/− SEM, Pressure Sensor data from nulliparous n = 5, primiparous n = 6, multiparous n = 5 ewes/group. **p < 0.01, ***p < 0.001, ****p < 0.0001.

Ovine vaginal wall displacement or tissue laxity was measured using a modified human POP-Q^32^ to provide a clinical measure of vaginal wall weakness^18^. Our modified “POP-Q” measures the distance (cm) vaginal tissue can be displaced using traction, with −3 indicating no displacement and +3 maximal displacement with respect to the muco-cutaneous junction or urethra^16^. Ovine vaginal length was also measured for nulliparous (11.3 ± 0.4 cm), primiparous (13.0 ± 0.5 cm) and multiparous (14.3 ± 0.3 cm) ewes. Nulliparous ewes showed no vaginal displacement at the three “POP-Q” points assessed; Aa, Ap and Ba, generating baseline measurements of −3 for each point (Fig. 1A). Primiparous ewes showed no displacement at Aa and Ba, but more laxity at Ap compared to nulliparous (p = 0.0026). In contrast, multiparous ewes showed significantly more vaginal displacement at all 3 “POP-Q” points compared to nulliparous (Ap, p < 0.001; Aa, p = 0.0011; Ba, p < 0.001) and primiparous ewes (Aa, p = 0.001; Ba, p < 0.001) (Fig. 1A). The genital hiatus (Gh, Fig. 1A), a measure of the pelvic floor muscle support was significantly larger for primiparous compared with nulliparous ewes (p = 0.0065). The perineal body (PB) was reduced in multiparous ewes, compared to nulliparous (p = 0.001) (Fig. 1A). Ovine vaginal wall weakness was also measured with a novel pressure sensor device^15^, which has eight fibre optic pressure sensors inserted 10 mm apart on each of the anterior and posterior blades of a modified speculum that enables the measurement of distributed pressure along both the posterior and anterior vaginal wall (Fig. 1B). Multiparous vaginal walls exerted significantly lower pressure than that of the nulliparous vagina in sensors 6–7 measurements, equivalent to point Ap (p = 0.0053) (Fig. 1B), while primiparous exhibited lower pressure in sensors 1–3 for the equivalent Ba region (p = 0.003) (Fig. 1B) measurements. No difference was found in vaginal pressure at sensors 6–7 of the lower blade (equivalent to “POP-Q” point Aa) for the three ewe groups (Fig. 1B). There was strong positive correlation between the “POP-Q” Ap measurement and Ap pressure (r = 0.70, p = 0.003), but not for points on the anterior wall, Aa or Ba (Table 1).

### Ewe vaginal wall structure changes with parity

**Figure 2:**
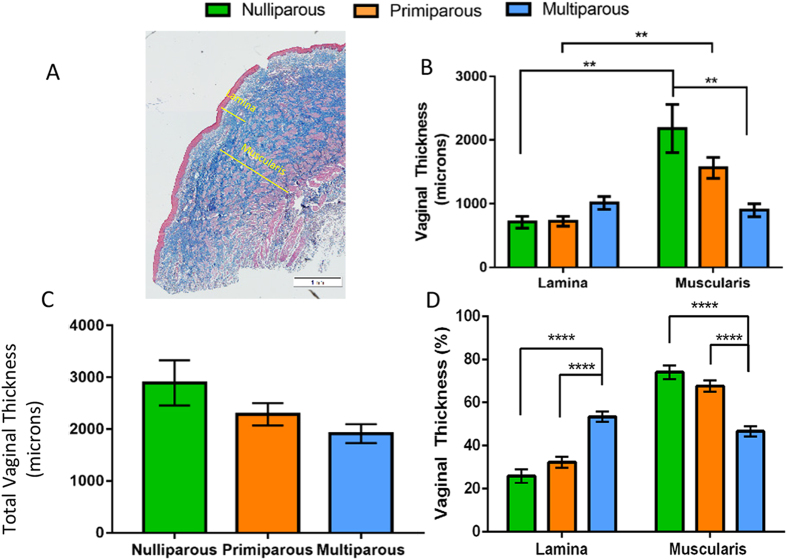
Dimensions of the ovine vaginal wall. (**A**) Mason’s Trichrome Staining showing measurement of lamina propria and muscularis (yellow lines). Vaginal thickness of the (**B**) lamina propria and muscularis and (**C**) the total vaginal wall thickness. (**D**) lamina propria and muscularis as percent of vaginal wall depth. Scale Bar is 1 mm. Data are mean +/− SEM from nulliparous n = 6, primiparous and multiparous n = 8 ewes/group. **p < 0.01, ***p < 0.001, ****p < 0.0001.

Masson’s trichrome staining of the ovine vagina showed the typical layered structure, with a lamina propria beneath the epithelium and a deeper muscularis layer (Fig. 2A). The thickness of the lamina propria was similar between ewe groups; however the muscularis was significantly thinner in the multiparous, compared with the ovine vaginal wall (p = 0.0018) (Fig. 2B). The thickness of the muscularis was greater than the lamina propria in the nulliparous (p = 0.0037) and primiparous (p = 0.0084) ovine vagina, while these regions had similar thickness in the multiparous ewes (Fig. 2B). When the lamina propria and muscularis were combined to determine the total thickness of each wall, no significant differences were observed between all three ewe groups (Fig. 2C). However, when these regions are reported as percentage of total wall thickness, the lamina propria was ∼50% thicker in the multiparous, compared with nulliparous and primiparous tissues (p < 0.001 and p < 0.001, respectively) (Fig. 2D). In contrast, the % muscularis of the multiparous ewes was significantly less than the nulliparous and primiparous ewes (p < 0.001, p < 0.001 respectively) (Fig. 2D). There was a strong negative correlation between “POP-Q” Aa and Ap and the muscularis thickness (Aa, r = 0.61, p = 0.002; Ap, r = 0.75, p < 0.001), indicating that the thin muscularis found in the multiparous ovine vagina is directly associated with vaginal wall weakness, particularly in the posterior compartment (Table 1).

### Collagen content and organisation differs little with parity

**Figure 3:**
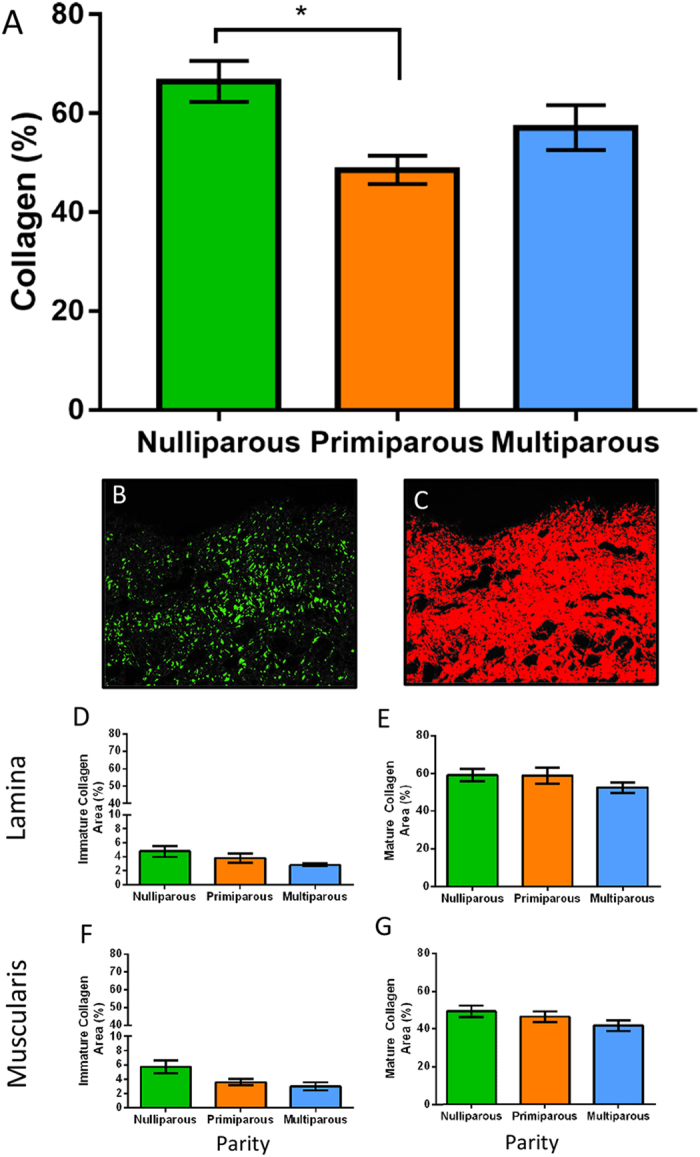
Collagen in the ovine vaginal wall. Biochemical analysis by hydroxyproline for (**A**) total collagen content as percent of dry weight. Birefringence images of Sirius Red stained tissues showing (**B**) green immature fibrils, and (**C**) red mature fibrils in the lamina propria. (**D**) immature and (**E**) mature collagen within the lamina propria of nulliparous (green bars), primiparous (orange bars) and multiparous (blue bars) ewes. (**F**) immature and (**G**) mature collagen within the muscularis. Data are mean+/− SEM from nulliparous n = 6, primiparous and multiparous n = 8 ewes/group.

Total collagen content of ovine vaginal tissue was measured biochemically using a hydroxyproline collagen assay, which showed significantly less collagen between nulliparous and primiparous tissues (Fig. 3A). Similarly, multiparous vaginal tissues had less collagen but this was not significant (Fig. 3A). Sirius Red birefringence was used to assess collagen organisation in the two layers of the ovine vagina (Fig. 3B,C). The percent area of both immature and mature collagen fibrils in the three ewe groups were similar in the lamina propria (Fig. 3D,E) and muscularis (Fig. 3F,G). There was a significant negative correlation between the muscularis immature collagen and the Ba pressure sensor measurement (r = 0.70, p = 0.002). However, there were no other significant correlations (Table 1).

### Parity has no effect on smooth muscle content of the ovine vagina

**Figure 4:**
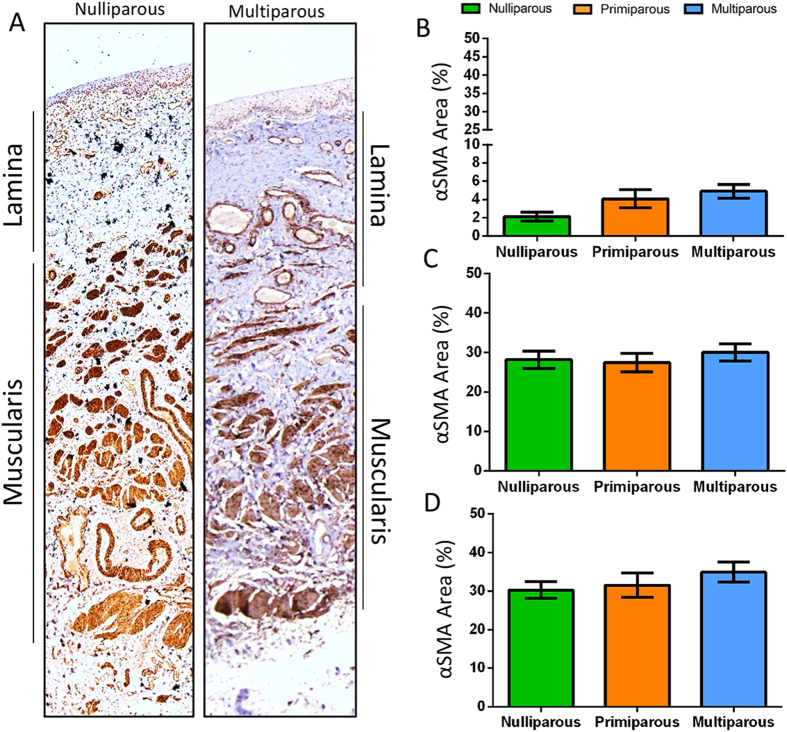
Smooth Muscle Content in the ovine vaginal wall by αSMA Immunohistochemistry. Images showing αSMA positive blood vessel vascularity of the ovine lamina propria of the vagina in **(A**) nulliparous and multiparous ewes. Quantification by image analysis of aSMA percent of the (**B**) lamina propria, (**C**) muscularis and the (**D**) total smooth muscle in vaginal tissue. Data are mean +/− SEM from nulliparous n = 6, primiparous and multiparous n = 8 ewes/group.

Alpha Smooth Muscle Actin (αSMA) immunostaining showed the bilayer arrangement of smooth muscle bundles in the muscularis of the ovine vagina (Fig. 4A). In the lamina propria, smooth muscle cells in the blood vessels were more prominent in multiparous tissue than nulliparous (Fig. 4A). The percentage area of smooth muscle as assessed by αSMA immunostaining was similar between the ewe groups in the lamina propria (Fig. 4B) and muscularis (Fig. 4C). Total αSMA percent area also showed no difference between ewe groups (Fig. 4D). Finally, there was no significant correlation between “POP-Q” measurements and % area of αSMA of the ovine vaginal wall compartments (Table 1).

### Elastic fibre content increases with parity in the ovine vagina

**Figure 5:**
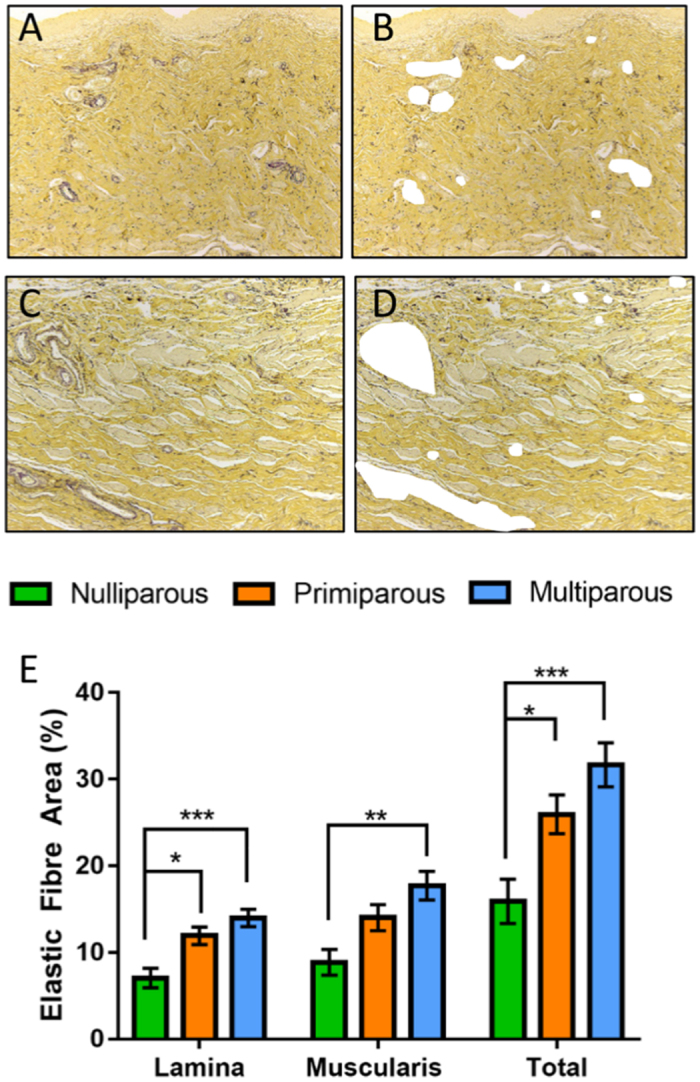
Elastic fibre content of ovine vaginal wall. Shown as black fibres in Hart’s-stained tissue in representative images of multiparous (**A**) lamina propria with (**B**) blood vessels removed from analysis and in the (**C**) muscularis with (**D**) blood vessels removed from analysis. (**E**) Graph showing the image analysis quantification of elastic fibre area in tissue images with blood vessels electronically removed. n = 6, primiparous and multiparous n = 8 vaginal tissues/group. Data are mean +/− SEM, *p < 0.05, **p < 0.01, ***p < 0.001.

Black-stained elastic fibres were predominant within both the lamina propria and muscularis of the multiparous (Fig. 5A,C) compared to the respective regions of the nulliparous vaginal wall. The elastic fibre content (%) with blood vessel contribution removed electronically (Fig. 5B,D) was increased in the lamina propria of primiparous and multiparous tissue compared to nulliparous (p = 0.0126, p < 0.001, respectively) (Fig. 5E). The vaginal muscularis of multiparous ewes also contained significantly greater elastic fibres than nulliparous ewes (p = 0.003) (Fig. 5E). Total elastic fibres in the entire vaginal wall was greater for primiparous and multiparous ewes compared with nulliparous (p = 0.0296, p < 0.001 respectively) (Fig. 5E). There was a positive correlation between the “POP-Q” measurement and the percent of elastic fibres within the lamina propria (Ap, r = 0.81, p < 0.001; Aa r = 0.67, p = 0.001) and the muscularis (Ap, r = 0.65, p = 0.001; Aa r = 0.6, p = 0.003). These correlations suggest that increased elastic fibres in the lamina propria and muscularis tissue may be associated with vaginal weakness.

### Tensile strength and linear stiffness decreases with parity in the ovine vagina

**Figure 6:**
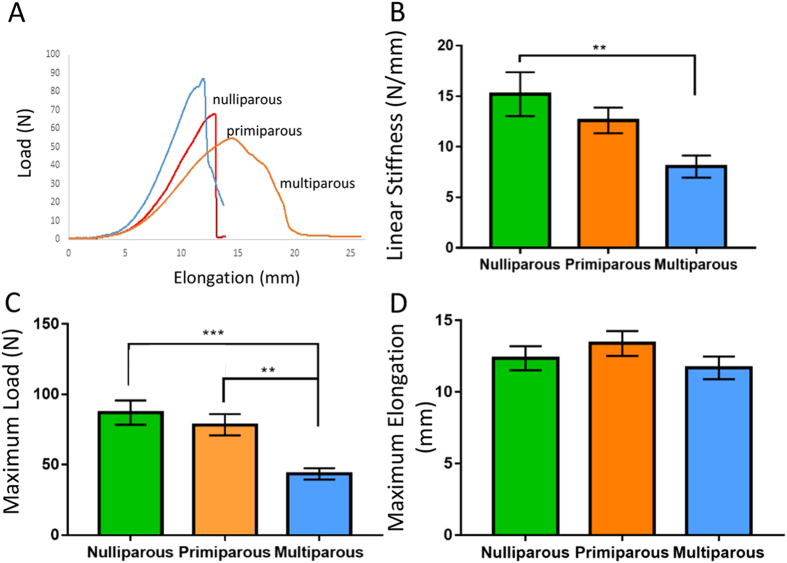
Biomechanical analysis of ovine vaginal tissue. Using biaxial tensile testing (**A**) representative load-elongation curves from nulliparous (blue), primiparous (red) and multiparous (orange) vaginal wall. (**B**) Linear stiffness, (**C**) Max Load and (**D**) Maximum Elongation. Data are mean +/− SEM from nulliparous n = 6, primiparous and multiparous n = 8 ewes/group. **p < 0.01, ***p < 0.001.

Biomechanical properties of the ovine vaginal wall were assessed by biaxial tensiometry, using the ball burst method. Typical load (N) versus elongation (mm) curves suggested that multiparous tissue (orange) exhibited reduced maximum load-bearing ability compared to nulliparous (blue) and primiparous (red) tissue (Fig. 6A). Multiparous vaginal tissue exhibited significantly lower linear region stiffness (Fig. 6B) than nulliparous tissue (p = 0.0093) and was significantly weaker than both the nulliparous (p = 0.0008) and primiparous (p = 0.0031) ewes with a lower breaking load (Fig. 6C). Maximum elongation of vaginal tissue was also measured, but no differences were observed between the three groups (Fig. 6D). “POP-Q” measurement were negatively correlated with Ap and Aa measurements of maximum load (Ap, r = 0.77, p < 0.001; Aa, r = 0.57, p = 0.005), (Table 1). Maximum load also negatively correlated with the Ap pressure sensor measurement (r = 0.74, p = 0.001) (Table 1). Linear stiffness and maximum load results also correlated (r = 0.6, p = 0.002) and there was also a strong correlation of maximum load with thickness of the muscularis (r = 0.7, p < 0.001) (Table 1). Together these correlations indicate vaginal weakness detected by a clinical measurement and that changes in support measured by the pressure sensor are associated with a thin muscularis with increased elastic fibre content and decreased biomechanical strength of ovine vaginal tissue, particularly in the posterior vaginal wall.

## Discussion

In our study we have shown that the ovine vaginal wall undergoes dynamic changes in physiological, structural, histological and physical properties as a result of increasing parity, with the greatest effect observed in multiparous ewes. We first observed significant differences in tissue displacement and pressure between parous groups using both modified “POP-Q” measurement and a new pressure sensor. Specifically, the multiparous vagina becomes thinner due to a loss of muscularis tissue, though density of the remaining smooth muscle is similar to both nulliparous and primiparous ewes. The elastic fibre content was greater within the primiparous and multiparous vagina muscularis and percent collagen is reduced in primiparous compared to nulliparous ewes. The vagina was also weaker and less stiff in multiparous ewes, compared to nulliparous and primiparous ewes, suggesting that the loss of muscle mass rather than elastic fibres is a significant contributor to higher “POP-Q” measurements and weaker biomechanical measurements. For the first time, our extensive correlation analysis shows a positive relationship between thickness of the vaginal muscularis and its mechanical strength, as well as a negative relationship between the ECM elastic fibre content and vaginal wall strength in the distal region of the anterior and posterior ovine vaginal wall.

Our correlative data suggests that the muscularis thins with increasing parity resulting in a disproportionately thicker, well vascularised lamina propria, which is associated with vaginal wall weakness, as measured by both clinical and biomechanical methods. Increased elastic fibre content in the weakened multiparous ovine vagina may be a compensatory mechanism for loss of muscularis. Our data also suggests that the decreased thickness of the muscularis may be a significant contributor to loss of vaginal wall strength and stiffness and that increasing parity has a considerable impact on this change in tissue compartment of the ovine vaginal wall. The similar percentage area of vaginal αSMA staining in the three groups of ewes suggests that the density of the muscle bundles is not altered in multiparous ewes. Rather, it appears that there is a loss of absolute levels of smooth muscle in the multiparous ewe vagina that may account for muscularis thinning. The total vaginal wall itself is not significantly thinner than that of nulliparous ewes, although there is a trend towards vaginal thinning from nulliparous to primiparous to multiparous ewes.

While significantly lower total collagen content was observed in the primiparous tissue than nulliparous using a biochemical assay, this was not replicated in our histological analysis using Sirius red birefringence, which may be less sensitive as it only measures collagen fibril organisation. Our data suggests that the nulliparous vaginal tissue contains more total collagen but has no relationship with ewe parity. As there was also no correlation observed between the collagen content and any other measurement, any effect this may have on the biomechanical properties and areas of weakness of the vaginal tissue remains unclear. Increased elastic fibre synthesis may have compensated for the loss of smooth muscle in the multiparous vagina. This increased elastic fibre content may account for the decreased tissue biomechanical strength and stiffness, along with the loss of muscularis, may account for the loss of tensile strength in multiparous vaginal tissue, compared to nulliparous and primiparous ewes. This was suggested by our “POP-Q” correlation results. Specifically, the muscularis elastic fibre content correlated positively with Ap and Aa measurements. Though a direct relationship requires further investigation, we are confident that a relationship exists due to our use of the Benjamini-Hochberg false discovery rate correction to calculate a stringent significance level of P < 0.0051, for which significance was met for only 15 out of a possible 141 correlations. Additionally, our previously reported POP-Q measurements in ewes had good-to-excellent intraclass correlation coefficients for Aa and Ap, but weaker for the Ba point^16^. In the present study, our correlations between POP-Q points Aa and Ap and histological and biomechanical data yielded very small P values in contrast to correlations with Ba providing further confidence in the accuracy of their measurement. Together these correlations may indicate POP susceptibility in the multiparous ewes. When considered as a whole, this suggests that the muscularis plays an important role in protecting the vaginal wall against herniation from pelvic organs, and its size reduction with accompanying smooth muscle and ECM alteration from repeated birth-induced injury may leave the vaginal wall vulnerable to prolapse. Our results support findings in the vaginal wall of multiparous menopausal women who are more susceptible to pelvic organ prolapse^31^,^33^,^34^,^35^,^36^.

An earlier smaller study in ewes showed the multiparous muscularis was thicker than the nulliparous^18^. It also showed greater collagen content and lower elastic tissue associated proteins (ETAP) measured biochemically. However, these differences may be due to different methodologies and sample sizes used in these studies. The current study used greater sample sizes for each of the groups. It also employed histological and biochemical methods for assessing elastic fibre and total collagen content, quantifying immature and mature collagen by Sirius Red birefringence in contrast to SDS-PAGE to assess collagens type I and III^18^. Our histological measure of vaginal wall thickness was from multiple measurements per region in each sample rather than a single measurement reported in the earlier study. The limited correlation observed between conventional “POP-Q” measurement and the novel pressure sensor device indicate further refinement is needed for the device, with particular attention to the distinction between tissue displacement and vaginal wall pressure as predictors of POP vulnerability. However, this novel device holds value in that it is capable of measuring alterations in pressure along the lengths of the anterior and posterior vaginal wall, instead of a single measurement for both. This may be achieved by automating dilation of the speculum to minimise movement artefact and determining the maximum rate of pressure change versus dilation to accommodate variations in vaginal dimensions that may be associated with parity or vaginal weakness^15^.

The results of our study align with the findings of others investigating the relationship between tissue composition and pelvic organ prolapse in other animal models. Our observations on collagen align with those performed in prolapsed tissue of rhesus macaques, where different collagen types and their ratio was not different to nulliparous controls, although there was less collagen alignment as observed under a polarising microscope^37^. Parous macaques also exhibited inferior biomechanical properties similar to our study. Smooth muscle is also significantly decreased due to increased apoptosis in human vaginal tissue obtained from women with POP^30^,^31^. Similar to the current study, the smooth muscle content was not diminished but rather the area the smooth muscle occupied had decreased^31^. Previous studies have reported that vaginal smooth muscle content of women with POP was diminished and disorganised^28^,^29^. However, in our study, we did not observe a disorganised muscularis in multiparous ewes, but rather a thinning of this entire layer, suggesting a unique adaptation in the ovine model not seen in parous women with POP. While our image analysis revealed a thinner muscularis in multiparous than in nulliparous ewes, we were unable to determine whether this was due to smaller smooth muscle cells or a reduction in smooth muscle cell number. Nevertheless, our study provides evidence that the net pathophysiological response of the altered vaginal muscularis is likely very similar in ewes and women.

The current understanding of elastic fibre synthesis within the vaginal wall, before and after child delivery, is limited and requires further investigation. As has been observed in Fibulin 5^−/−^ knockout mice, elastic fibre deficiency contributes to the structural weakness of the vaginal wall that leads to POP. In contrast, the weakened ovine vaginal wall of the primiparous and multiparous ewes at Ap, as evidenced by POP-Q and pressure sensor measurements, showed increased levels of elastic fibre content compared to nulliparous ewes. It is possible that elastic fibres are synthesised within the vaginal wall after lamb delivery as a compensatory mechanism for the loss of smooth muscle. Increased elastic fibre content of vaginal tissue from the prolapse site has also been observed women^10^. However, the precise relationship between increased elastic fibre content, vaginal wall weakness and POP requires further research.

Indeed, that our results aligned with those that used humans and macaques as models supports our selection of ewes as an investigational model. Our study also supports a recent report comparing the mechanical properties of ewe vagina in non-human primates and rodent models where parity had a negative impact on mechanical integrity^38^. Though further comparative studies between these species are warranted, the similarities already identified suggests that ewes can be used for further investigations. The ewe can serve as a model for measuring damage caused by the first vaginal delivery, known to be a major contributor to future POP^1^. Our observations in multiparous ewes suggest they may be a suitable model for assessing new treatments for POP, such as cell-based therapies^11^,^12^.

Our data indicate the muscularis as a key structural determinant of biomechanical strength of the vaginal wall. However, a limitation of the biaxial tensiometry used in this study (“ballburst” test), is that a steel rod may not exert equivalent forces of a pelvic organ, with different shape and hardness. However, the vaginal wall is subject to high intra-abdominal pressures transmitted through pelvic organs pressing on the vaginal wall^39^. Vaginal tissue is anisotropic^19^,^40^, thus biaxial testing is currently the most equivalent test available. It is superior to uniaxial biomechanical testing as this only measures properties in one direction, which does not represent the physiologic environment, in which strain is placed on the vaginal wall in all directions^18^,^26^,^41^.

Limitations of our study include the lack of hormone measurement to determine oestrus cycle stage of the ewes, relatively small group size and lack of longitudinal measurements of POP-Q points or pressure sensor readings. Although the majority of our samples were from ewes in the follicular phase of the oestrus cycle, it is unknown whether the luteal stage would have affected our results. Given that the greatest damage to the vaginal wall occurs in women during their first vaginal birth and that the risk of POP rises incrementally with subsequent births^42^,^43^,^44^ we expected to see greatest changes in these measurements between nulliparous and primiparous ewes compared to those between the primiparous and multiparous. While our small group sizes may have contributed to minimal changes observed in primiparous ewes at the POP-Q points Aa and Ba compared with nulliparous, we also showed trends toward greater changes for GH and PB than between primparous and multiparous and for Ap and Ba using the pressure sensor device. This suggests that the pressure sensor device may be more sensitive in detecting vaginal wall weakness than the relatively inexact POP-Q measurements and therefore may have value in assessing women with POP. Similarly, our small group size and lack of longitudinal data may have contributed to our observation of the greatest effect on muscularis and vaginal thickness in multiparous rather than primiparous ewes. Our data also suggests that the Ba measurement may not be as robust as other POP-Q points as significant correlations (P < 0.0051) were not observed at this point for muscularis thickness, elastic fibre content or maximum load. This may be due to the urethral opening positioned in the anterior vaginal wall, which is not the case in women and may explain why POP, while very common in the Ba position in women, is not in the ovine model^43^. Since vaginal surgical procedures in the ovine model are performed in the Ap region^45^,^46^ rather than the anterior wall because of the urethra, we believe that the POP-Q and pressure sensor values at this point will provide a useful monitoring parameter.

In conclusion, we have observed vaginal muscularis thinning following multiple births in an ovine model which may significantly contribute to vaginal wall weakness and potential susceptibility to POP. In particular we identified that loss of smooth muscle in the vaginal muscularis is a key factor in vaginal muscularis thinning, and is associated with loss of strength and stiffness resulting in vaginal wall weakness in multiparous ewes. We contend that it is the thinner muscularis of the multiparous vaginal wall that makes it vulnerable to rises in intra-abdominal pressure and allows the herniation of the pelvic organs, resulting in POP. Our data contributes to the understanding of the effect of parity on ovine vaginal tissue structure and mechanical properties, which will inform future studies that may use this large pre-clinical animal model for cell-based therapies for POP.

## Methods

### Ethics and Animals

Experimental procedures and animal husbandry were approved by the Monash Medical Centre Animal Ethics Committee A in accordance with the ethical guidelines of the National Health and Medical Research Council (NHMRC) of Australian Code for the Care and Use of Animals for Scientific Purposes 8^th^ Edition. Border Leicester Merino (BLM) ewes were housed in the Monash Animal Service. Ewes were selected on their birth history; nulliparous (having never been pregnant, aged 2 years, n = 6), primiparous (having had one pregnancy, aged 3–4 years, n = 8) and multiparous (having more than one previous pregnancy, aged 4–5 years, n = 8) who had undergone zero, one and multiple lamb deliveries, respectively, with the last lamb delivered 12 months prior.

### Modified Pelvic Organ Prolapse Quantification (“POP-Q”) measurements

“POP-Q” measurements were undertaken by gynaecologists (NY, AR) in conscious ewes as previously described^18^. Briefly manual traction with forceps on the vaginal tissue in the Aa, Ba, Ap regions using the urethra as a reference point for Ba (proximal anterior) and mucocutaneous junction for Aa (distal anterior) and Ap (distal posterior), rather than the hymen as performed in women. The intraclass correlation coefficients for the POPQ measures Aa, Ap and Ba were 0.75, 0.83 and 0.64, respectively, and interclass correlation coeffeicents 0.73, 0.74 and 0.58 respectively. Vaginal length of each ewe was also recorded.

### Vaginal pressure measurements

Immediately following “POP-Q” measurements, a modified speculum fitted with 8 fibre optic pressures sensors 10 mm apart on each parallel blade was inserted into the ovine vagina and pressure measurements recorded at 10 mm dilation following a maximal dilation of 20 mm. Mean pressures (mm Hg) at sensor 1–3, equivalent to Ba (anterior blade, lower proximal toward the cervix), and sensors 6–7 on each blade, equivalent to Aa (anterior blade, lower distal toward introitus) and Ap (upper posterior blade, distal toward introitus) were reported.

### Tissues

Following euthanasia, the complete vaginal tract was removed, trimmed and incised in a longitudinal manner adjacent to the urethra (anterior wall) from the muco-cutaneous junction to the cervix (Supplemental Fig. 1) and dissected and frozen at −20 C for biomechanical analyses. Due to narrowing of the ewe vagina at the apical end, the 4 cm × 4 cm tissue required for biomechanical testing was taken from the mid-region of the posterior wall. Previous studies have demonstrated similarity in the biochemical and biomechanical properties of the lower-and-mid regions of the ovine vaginal wall^41^. To avoid the hymnal ring, tissue adjacent to the piece for biomechanical analysis was excised and fixed in 10% formalin, paraffin embedded and 5 μm sections were stained with Haemotoxylin and Eosin (H&E), Masson’s Trichrome and Hart’s elastic fibre stain in the Monash Histology Platform (MHP) facility. Additionally, ovaries were removed from each animal in order to determine the stage of oestrus cycle; 4/6 nulliparous, 7/8 primiparous and 8/8 multiparous ewes were in the follicular stage of the oestrus cycle.

### Vaginal wall thickness

Massons Trichrome-stained sections were examined under an Olympus BX61 light microscope and three high resolution images per sample of the entire vaginal wall acquired using Olympus cellSense software. Both the lamina propria and muscularis were measured in microns (n = 3/region) and replicates averaged for calculating mean/ewe group. The total vaginal wall thickness was calculated by adding the length of the 2 regions and the relative percentage of each region to total vaginal wall thickness was calculated.

### α-Smooth muscle actin immunohistochemistry and image analysis

Sections were dewaxed, rehydrated, then protein block (Dako Glostrup, Denmark) was applied for 30 min at RT. After three washes in PBS, sections were incubated with mouse anti-human α-smooth muscle actin (αSMA) antibody (Dako) for one hr at 37 °C at 1:50 dilution (71 μg/1 ml). Mouse IgG1 isotype control antibody (Dako) was used as the negative control at the same concentration. Sections were washed 3 times with PBS/2% BSA followed by anti-mouse secondary antibody (Dako) for 30 mins at RT, washed in PBS and chromogen added (3,3′-diaminobenzidine) (Sigma-Aldrich, St. Louis USA) for 3 min as previously published^18^. Slides were mounted in DPX (Sigma-Aldrich). Three images (10X magnification) were taken each of the lamina propria and muscularis regions using an Olympus BX61 light microscope and Olympus cellSens software and analysed using ImageJ software. Each image was color deconvoluted from haematoxylin and DAB staining, then converted into binary colour, with pixels above a threshold intensity of 20 considered as 1 (αSMA+) and everything else 0. The area of brown-stained smooth muscle in each region was then measured as a percentage of the area of interest, then averaged for the three replicates for each ovine sample and then averaged for each of the 3 groups.

### Collagen analysis

Tissue sections were stained with Picro-Sirius Red using previously established protocols^47^. Briefly, slides were stained in Weigert’s haematoxylin for 8 minutes, then washed in tap water for 10 minutes before staining in 0.1% Picro-Sirius red for one hour then washed in two changes of acidified water (0.5% glacial acetic acid, pH 3). Slides were allowed to air-dry, rinsed twice in Histoclear and mounted in DPX. Three images at 10X magnification were taken of the lamina propria and muscularis regions using an Olympus BX61 light microscope equipped with a polarising filter (Olympus T2 U-ANT and U-POT) using cellSens software and an Olympus DP80 camera to identify birefringent Sirius red-stained collagen fibres. Images were examined using ImageJ software and separated into base colors red and green. An intensity threshold was set that converted any green or red pixel into a single colour with a count of 1, while a black pixel was considered 0. The red and green birefringent collagen fibres (Fig. 3A–C) were measured as percentage area of both the lamina propria and muscularis for three images/region. These replicates were averaged for each ovine sample and used to obtain means for each group.

### Hydroxyproline Assay for Collagen

Supernatants from digested samples were immersed in 5 ml of 6 M HCl and 2 ml edible oil under nitrogen for 4 hours at 11.5 C, then transferred to a desiccator overnight. The solution beneath the oil was extracted and evaporated at 70 C. The precipitate was weighed in 4 ml of distilled water, then 0.5 ml of 0.05 M Chloramine T solution was added to each sample for 30 min at room temperature (RT). The reaction was continued by addition of 0.5 ml of 3.15 M Perchloric Acid Solution for 10 minutes at RT, followed by adding 0.5 ml 10% Paradimethylaminobenzaldehyde solution and incubated for 40 minutes at 60 C. Absorbance was read at A560 nm using distilled water as a blank. A standard curve using L-hydroxyproline (0–10 ug/ml (Sigma)) was used to ascertain the collagen content of each sample. Total collagen was calculated using hydroxyproline: collagen ratio of 0.143:1.

### Elastic fibre analysis

Dewaxed and dehydrated paraffin sections were stained by Hart’s method at the Monash Histology Platform. Briefly, sections were immersed in 0.25% potassium permanganate for 5 minutes, then rinsed in distilled water twice for 30 seconds each, and placed in 5% oxalic acid for 3 seconds before rinsing in tap water, then distilled water, twice for 30 seconds each followed by 10% Resorcin (w/v distilled water) Fuchsin (2% w/v distilled water) solution in acidified 70% ethanol overnight. Slides were again rinsed in tap then distilled water three times, each for 30 seconds and counterstained in 0.25% tartrazine in saturated picric acid for 3 minutes, dehydrated, cleared and mounted with DPX. Three images (10X magnification) were taken of the lamina propria and muscularis regions using an Olympus BX61 light microscope. Blood vessels in the images were outlined and removed electronically before a custom ImageJ macro was used to separate the image into black (elastic fibre) and yellow (background tissue) stained components, with a threshold of 20 considered as 1 (elastic fibre+) and everything else 0 and then generated the area percentage of elastic fibre and yellow-stained tissue in the area of interest (Fig. 5). The percentage area of elastic fibre was then divided by the area percentage of tissue to produce the percentage elastic fibre/region, then averaged for three replicates from each ovine sample and the average for each group then calculated.

### Biomechanical analysis of ovine vaginal tissue

Frozen tissues were thawed overnight at 4 C and tested within 24 hr. Freeze-thawing vaginal tissue does not alter its mechanical properties and is more reliable as the same conditions are used for each specimen^48^. Samples (∼30 × 30 mm) were excised from the explanted sample and kept moist using PBS until testing. The biaxial tensile properties of the tissue were measured using a ball burst test method using an Instron Tensile Tester (5557; Instron Corp, MA) with a load cell of 100N. Samples were secured between 2 embossed metal plates, both with an aperture of 15 mm (for penetration of the steel rod). Rubber sheeting was used to avoid sample slippage during testing. A rounded steel rod (10 mm diameter) was pushed through the tissue sample at a crosshead speed of 20 mm/min to break. Load-elongation curves were plotted from the generated data (Fig. 6A) and from these curves stiffness (N/mm) in the region of high stiffness (linear region), maximum load (N) and maximum elongation were calculated.

### Statistical analysis

All data were assessed for normality using Shapiro-Wilk or Kolmogorov-Smirnov tests if the sample size was too small for Shapiro-Wilk test. Data were normally distributed. Differences in POP-Q scores, pressure sensor data, histological and biomechanical data by parity group (nulliparous, primiparous and parous) were assessed using a 1-Way ANOVA with Tukey’s multiple comparison post hoc test. The correlation between the “POP-Q”, biomechanical data and pressure sensor with histological parameters was assessed using the Pearson’s correlations coefficient. Due to the multiple testing we performed a Benjamini-Hochberg false discovery rate calculation. All correlation p values < 0.0051 (2-tailed) were considered statistically significant. All analyses were undertaken using GraphPad Prism 6 for Windows 7, (GraphPad Software, La Jolla California USA) or the SPSS statistical package (SPSS 23, IBM Corp, Armonk, New York, USA).

## Additional Information

**How to cite this article:** Emmerson, S. *et al*. Ovine multiparity is associated with diminished vaginal muscularis, increased elastic fibres and vaginal wall weakness: implication for pelvic organ prolapse. *Sci. Rep.* **7**, 45709; doi: 10.1038/srep45709 (2017).

**Publisher's note:** Springer Nature remains neutral with regard to jurisdictional claims in published maps and institutional affiliations.

## Supplementary Material

Supplementary Information

## Figures and Tables

**Table 1:** Correlation analyses of ovine vagina between POP-Q, pressure sensor, histological and biomechanical parameters.

Variable	Variable Correlation R-coefficients								
	Ap	Aa	Ba	Linear Stiffness	Max Load	Max Elongation	Ap Pressure	Aa Pressure	Ba Pressure
Ap Pressure	**0.7, 0.003**	0.6, 0.251	0.47, 0.063	0.280	0.74, 0.001	0.13, 0.634			
Aa Pressure	0.17, 0.518	0.04, 0.878	0.004, 0.989	0.32, 0.216	0.5, 0.041	0.08, 0.755			
Ba Pressure	0.51, 0.037	0.3, 0.244	0.27, 0.294	0.071, 0.790	0.37, 0.146	0.04, 0.859			
Lamina Propria	0.1, 0.66	0.15, 0.502	0.35, 0.106	0.33, 0.131	0.3, 0.171	0.29, 0.188	0.14, 0.602	0.11, 0.681	0.12, 0.634
Muscularis	0.75, <0.001	0.61, 0.002	0.54, 0.01	0.44, 0.038	0.7, <0.001	0.13, 0.564	0.58, 0.017	0.1, 0.699	0.42, 0.089
Collagen Lamina Immature	0.52, 0.014	0.45, 0.038	0.39, 0.75	0.3, 0.166	0.44, 0.042	0.36, 0.095	0.34, 0.195	0.21, 0.414	0.32, 0.215
Collagen Lamina Mature	0.24 0.281	0.43, 0.043	0.31, 0.165	0.11, 0.628	0.09, 0.685	0.38, 0.082	0.001, 0.996	0.19, 0.465	0.01, 0.967
Collagen Muscularis Immature	0.53, 0.011	0.47, 0.028	0.32, 0.146	0.23, 0.293	0.51, 0.015	0.13, 0.547	0.45, 0.080	0.1, 0.703	0.67, 0.002
Collagen Muscularis Mature	0.36, 0.103	0.51, 0.015	0.36, 0.104	0.055, 0.821	0.22, 0.331	0.11, 0.608	0.055, 0.842	0.19, 0.468	0.52, 0.032
Collagen% of Dry Weight	0.092, 0.169	0.045, 0.342	0.001, 0.887	0.001, 0.883	0.020, 0.533	0.015, 0.591	0.089, 0.263	0.066, 0.32	0.207, 0.067
αSMA Lamina	0.43, 0.047	0.18, 0.458	0.26, 0.246	0.25, 0.253	0.4, 0.068	0.28, 0.208	0.21, 0.428	0.055, 0.845	0.38, 0.132
αSMA Muscularis	0.09, 0.691	0.044, 0.835	0.055, 0.811	0.25, 0.26	0.15, 0.51	0.48, 0.023	0.055, 0.88	0.21, 0.421	0.22, 0.795
Elastic Fibres Lamina	**0.81, <0.001**	**0.67, 0.001**	0.51, 0.15	0.36, 0.98	0.52, 0.014	0.14, 0.538	0.43, 0.97	0.01, 0.976	0.46, 0.06
Elastic Fibres Muscularis	**0.65, 0.001**	**0.6, 0.003**	0.49, 0.021	0.35, 0.109	0.49, 0.019	0.17, 0.438	0.34, 0.194	0.09, 0.74	0.52, 0.032
Linear Stiffness	0.54, 0.01	0.27, 0.226	0.37, 0.091		**0.6, 0.002**	0.09, 0.681	0.28, 0.293	0.32, 0.216	0.07, 0.0790
Max Load	0.74, <0.001	0.57, 0.005	0.54, 0.009	**0.6, 0.002**		0.14, 0.545	0.74, 0.001	0.5, 0.041	0.37, 0.146
Max Elongation	0.06, 0.795	0.22, 0.321	0.3, 0.175	0.09, 0.681	0.14, 0.545		0.13, 0.634	0.08, 0.755	0.04, 0.859

*Data are presented as r value, p value for each comparison. Benjamini-Hochberg procedure calculated a corrected significance value of p < 0.0051 to account for false discovery rate associated with multiple correlations. Values with underline are negative correlations, those in bold-underline were positively correlated.
