# Supplementary material for: Ovine multiparity is associated with diminished vaginal muscularis, increased elastic fibres and vaginal wall weakness: implication for pelvic organ prolapse
Source: Sci Rep. 2017 Apr 4;7:45709. doi: 10.1038/srep45709 (PMC5379562; doi:10.1038/srep45709)
Supplement: Supplementary Information [file srep45709-s1.pdf]

Ovine multiparity is associated with diminished vaginal muscularis, increased elastic fibres and vaginal wall weakness: implication for pelvic organ prolapse.

Stuart Emmerson, Natharnia Young, Anna Rosamilia, Luke Parkinson, Sharon L Edwards, Aditya V. Vashi, Miranda Davies-Tuck, Jacinta White, Kirstin Elgass, Camden Lo, John Arkwright, Jerome A Werkmeister, Caroline E Gargett

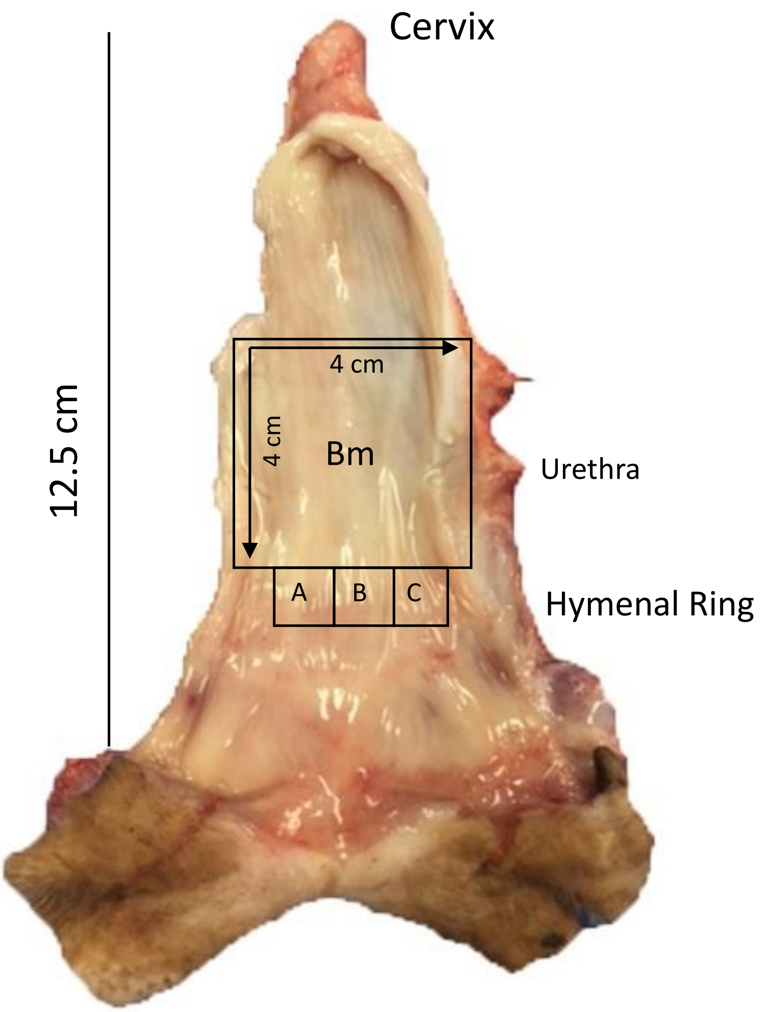

Supplemental Figure 1: Tissue Dissection
Tissue was dissected from vaginal tissue for **A)** Frozen Sections in OCT, **B)** paraffin embedding, **C)** biochemical analysis and **Bm)** Biomechanical analysis.
